# Prevalence of Histological Transformation in First-Line Osimertinib Non-Small Cell Lung Cancers: Case Series and Literature Review

**DOI:** 10.3390/ijms262110462

**Published:** 2025-10-28

**Authors:** Rebecca Sparavelli, Rossella Bruno, Alessandra Celi, Andrea Sbrana, Iacopo Petrini, Antonio Chella, Clara Ugolini, Greta Alì

**Affiliations:** 1 Surgical, Medical, Molecular, and Critical Care Pathology Department, University of Pisa, 56126 Pisa, Italy; rebecca.sparavelli@phd.unipi.it (R.S.); aled.celi1@gmail.com (A.C.); clara.ugolini@unipi.it (C.U.); 2 Unit of Pathological Anatomy, University Hospital of Pisa, 56126 Pisa, Italy; 3 Pneumo-Oncology Unit, Azienda Ospedaliero-Universitaria Pisana, 50134 Pisa, Italy; andreasbrana89@gmail.com (A.S.); anto.kell@tiscali.it (A.C.); 4 Department of Translational Research and of New Surgical and Medical Technologies, University of Pisa, 56126 Pisa, Italy; iacopo.petrini@gmail.com

**Keywords:** non-small cell lung cancer, adenocarcinoma, *EGFR*, acquired resistance, histological transformation, Osimertinib

## Abstract

Patients with metastatic lung adenocarcinoma (mADC) harboring *EGFR*-activating mutations can benefit from first-line Osimertinib, but acquired resistance inevitably occurs. Different resistance mechanisms, on- and off-target, have been described. Here, we evaluated the prevalence of phenotypic transformation as a resistance mechanism in a consecutive series of *EGFR*-mutated mADC, diagnosed at our institution, and on the basis of literature data. A consecutive 3-year series of non-small cell lung cancer (NSCLC) was reviewed according to histological and molecular characteristics. A total of 100 mADCs harboring *EGFR* exon-19 deletions (61 cases) and the p.(L858R) mutation (39 cases) were selected. All cases were treated by first-line Osimertinib. The prevalence and type of phenotypic transformation were evaluated in patients with available rebiopsy at the time of first-line progression. A total of 32 mADC patients underwent rebiopsy upon first-line Osimertinib progression, and 23 cases had *EGFR* exon-19 in-frame deletions and 9 p.(L858R) mutations. Four cases showed a phenotypic transformation after a median of 15 months from the start of Osimertinib treatment. All these cases harbored *EGFR* exon-19 deletions and *TP53* pathogenic mutations on diagnostic tumor tissues. Three cases switched to small cell lung cancer histology; in one case, a *MET* amplification was also detected on rebiopsy. One case changed to spindle cell carcinoma. All cases maintained the initial activating *EGFR alteration*. For three cases, liquid biopsy was performed at the time of progression: one was negative, one presented only an *EGFR* exon-19 deletion, and one presented only a *MET* amplification. In our study, phenotypic transformation had a considerable prevalence among *EGFR*-positive mADC patients treated by first-line Osimertinib. Different types of histological changes were detected as the only resistance mechanism except for one case with a simultaneously acquired *MET* amplification. Moreover, all cases harbored *TP53* alterations, influencing treatment response. Despite the usefulness of liquid biopsy, rebiopsy should be executed whenever possible. Indeed, it remains the only tool for assessing histological transformation, which greatly impacts prognosis and treatment decisions.

## 1. Introduction

Lung cancer is one of the main causes of cancer-related deaths worldwide [1]. Non-small cell lung cancer (NSCLC) accounts for 85–90% of all lung cancers, while small cell lung cancer (SCLC) only accounts for 10–15% [2]. According to its histopathological features, NSCLC can be divided into three major types: adenocarcinoma (ADC), squamous cell carcinoma (SCC), and large cell carcinoma. Less common types of NSCLC include large cell neuroendocrine carcinoma (LCNEC), sarcomatoid carcinoma, adenosquamous carcinoma, and salivary gland carcinoma [2]. ADC is the most common histotype, representing 50–60% of all NSCLCs. About 70% of lung ADC cases are diagnosed in the advanced/metastatic stages [3]. The identification and understanding of “driver mutations” in ADC led to the development of molecularly targeted therapies, particularly for advanced/metastatic tumors. Currently, several tyrosine kinase inhibitors (TKIs) are approved for the first or subsequent treatment lines of lung ADC, requiring the molecular testing of actionable alterations within the following genes: epidermal growth factor receptor (*EGFR*), anaplastic lymphoma kinase (*ALK*), B-Raf proto-oncogene (*BRAF*), ROS proto-oncogene 1, receptor tyrosine kinase (*ROS1*), ret proto-oncogene (*RET*), neurotrophic tyrosine receptor kinase (*NTRK1*, *NTRK2*, and *NTRK3*), MET proto-oncogene (*MET*), Kirsten rat sarcoma (*KRAS*), and human epidermal growth factor receptor 2 (*HER2*) [4]. Approximately 20–30% of lung ADC harbor driver mutations in the *EGFR* gene. *EGFR* encodes for a growth factor receptor that induces cell differentiation and proliferation through the activation of a tyrosine kinase signaling pathway [5,6]. About 90% of reported *EGFR* activating mutations are represented by in-frame deletions in exon 19 and the p.(L858R) point mutation in exon 21 [7].

Among resistance mechanisms, histological transformation is a process characterized by major remodeling of the cytoskeleton by the epithelial-to-mesenchymal transition process with a key role in tumor transformation, invasion, and metastasis. Particularly, phenotypic transformation from ADC to SCLC or SCC was reported in 2–15% of patients who progressed during treatment with Osimertinib [5,8,9].

This study aims to evaluate the prevalence of phenotypic transformation in a 3-year unselected consecutive series of metastatic *EGFR* mutated lung ADC diagnosed at our institution and treated by first-line Osimertinib. Moreover, literature data about histological transformation of NSCLCs after TKI treatment were reviewed.

### 1.1. Histological Transformation: A Literature Review

#### 1.1.1. Histological Transformation of *EGFR*-Mutated ADCs to SCLC

Histological transformation into SCLC is the most common type during TKI treatment [10]. SCLC is highly aggressive with a very poor prognosis accounting for 10–15% of all lung cancers [2]. Several studies reported that 3–14% of *EGFR*-mutated ADCs undergo phenotypic transformation into SCLC as a mechanism of resistance to EGFR-TKI (Table 1). The median transformation time from the start of treatment is about 18 months [11,12]. Histological examination and neuroendocrine markers evaluation on tumor tissue biopsy are crucial for transformed SCLC (tSCLC) diagnosis [13]. The molecular mechanisms behind this transformation are still unclear; however, two possible hypotheses have been suggested. According to the first one, transformation occurs by the trans-differentiation of primary adenocarcinoma cells into SCLC cells during EGFR-TKI treatment. Whereas in the second hypothesis, transformation occurs from pre-existing, dominant SCLC cells under the selection pressure of EGFR-TKIs [14]. Pre- and post-therapy samples were molecularly analyzed to better characterize this phenotypic change. For instance, it has been widely reported that tSCLC tumors often retain the original *EGFR*-activating mutation [14]. Another relevant issue is the potential impact of co-occurring gene mutations on phenotypic transformation. Among the most prevalent molecular mechanisms involved in NSCLC to SCLC transformation, there are tumor protein p53 (*TP53*) mutations, retinoblastoma 1 (*RB1*) loss, lack of EGFR expression and *MYC* amplification [15]. According to recent studies, concurrent *TP53* and *RB1* alterations are the most frequent in *EGFR*-mutated tSCLC [14,16,17]. To date, it has not been fully elucidated whether loss of function mutations in *RB1* and *TP53* are an early event in *EGFR*-mutant tumors or an acquired event occurring later in the histological change process [17]. Offin et al. hypothesized that *EGFR*-mutant lung cancers with *RB1/TP53* alterations can be associated with a high risk of SCLC transformation [18]. Studies focused on the Notch signal pathway, a key player in transdifferentiating processes, have shown that alterations in its regulation cause a loss of RB1. In the presence of *TP53* mutations, RB1 loss may be involved in neuroendocrine differentiation in non-neuroendocrine tumor tissues [15]. tSCLC is characterized by an unfavorable prognosis, and an interval of more than 12 months between NSCLC diagnosis and SCLC transformation has been associated with longer survival than cases with earlier SCLC transformations [19].

Despite the diagnosis of histological transformation requiring a rebiopsy, liquid biopsy provides an impressive amount of data, potentially correlating to the transformation process, even if no validated biomarkers are yet available [5].

#### 1.1.2. Histological Transformation of Pre- and Post-Therapy Samples—Mutated ADCs to SCC

Histological transformation from ADC to SCC is rare but may occur after treatment with EGFR-TKIs [26]. Pulmonary ADC and SCC have different characteristics regarding origin cells and tumor localization. Lung ADCs originate from type II pneumocytes or club cells, while lung SCCs derive from basal cells located underneath the trachea or bronchus epithelium. Moreover, ADCs are frequently located at distal bronchioles, whereas SCCs are often found at more proximal airways [27]. Squamous cell transformation (tSCC) has been reported in 1.1–14% of EGFR-positive ADCs, developing resistance to TKIs [28]. Song et al., in a retrospective cohort of 233 *EGFR*-mutated patients, showed that 11% of cases developed histological transformation, 42% were tSCC and transformation occurred 19–20 months after therapy start [29]. The most common mutations involved in tSCC are within *EGFR/RB1/TP53* genes; but also *APC/MED12/RBL2* alterations can contribute to this histological change [30]. Recent studies using a *KRAS* p.(G12D) mutation-positive cell line and a mouse model have indicated that the deletion of liver kinase B1 (LKB1) may be closely related to tSCC [31]. tSCC rarely responds to EGFR-TKI therapy, independently of its driver mutation, and it has a worse prognosis. The median post-transformation survival is about 12 months, while after SCC transformation, it is about 17 months [32].

#### 1.1.3. Histological Transformation of *EGFR*-Mutated ADCs to LCNET

LCNET is an uncommon subtype of malignant pulmonary tumor with a prevalence of 0.3–3% [33]. Histological transformation from *EGFR*-mutated ADC to LCNEC, as a mechanism of resistance to EGFR-TKI, is extremely rare (about 0.1% of patients) [34]. Despite its rarity, in all reported transformed cases, the initial *EGFR* activating mutation is retained, suggesting an origin from a previously present EGFR-dependent clone [35,36,37,38]. In tLCNET, EGFR protein expression appears to be suppressed, especially in cases with concurrent *RB1* and *TP53* mutations, as for tSCLC [37]. Although further studies are necessary, the detection of *RB1* and *TP53* co-occurring alterations in diagnostic specimens could be useful to better monitor tumor progression and clinical outcome. [37].

#### 1.1.4. Histological Transformation of EGFR-Mutated ADCs to Sarcomatoid Carcinoma

Sarcomatoid carcinoma is a rare subtype of NSCLC with a prevalence of about 0.4% [39]. The histological transformation from NSCLC to sarcomatoid carcinoma occurs in 2.5–4.8% of patients after failure of treatment with first/second and third-generation EGFR-TKIs [39]. *TP53* mutations, RB1 inactivation, *MET* over-expression, and PI3K/AKT/mTOR pathway activation are frequently observed [40]. The sarcomatous transformation represents the process whereby the neoplastic cell acquires mutations responsible for the epithelial–mesenchymal transition (EMT). Most of these mutations occur within genes encoding for transcriptional factors involved in regulatory pathways, such as zinc finger E-box binding homeobox (*ZEB1* and *ZEB2*) and snail family transcriptional repressor (*SNAI1* and *SNAl2*) [41]. Transformation into sarcomatoid carcinoma has an extremely poor prognosis. After transformation, EGFR-TKIs are not effective, even if the original *EGFR*-activating mutation is retained. In some cases, *MET* amplification is detected and may qualify patients for targeted therapy. In contrast, high PD-L1 expression levels (even > 80% in sarcoma) usually lead to immunotherapy and chemotherapy approaches [39,40].

## 2. Case Description

### 2.1. Study Cohort

From 2020 to 2022, one hundred patients with lung ADC harboring *EGFR* common mutations were diagnosed at our institution and included in this study. Sixty-one percent of patients were female and 39% were male. The median age was 68 years (range 34–91 years). Molecular analyses were performed at time of tumor diagnosis using biopsy sample in 61% of cases, cytological specimens in 36%, and surgical specimens in 3%, Approximately 81% of tumor specimen sites were located in the lung, while the remaining 19% were located in other tissues (6% lymph nodes; 4% liver; 5% bones; less than 4% on heart, brain, and diaphragm). Detailed clinicopathological features of the patients are reported in Supplementary Table S2.

### 2.2. Molecular Data

Overall, 61 out of 100 *EGFR*-mutated tumors harbored exon-19 deletion, while 39 cases carried the p.(L858R) point mutation. Three cases with the p.(L858R) mutation showed also additional *EGFR* alterations: one case had the p.(V834L) mutation in exon 21, one had the p.(A871G) mutation in exon 21, and one case had a p.(S768I) mutation in exon 20.

Details about *EGFR* alterations observed in the study cohort are summarized in Table 2.

### 2.3. Histological Transformation

**Figure 1:**
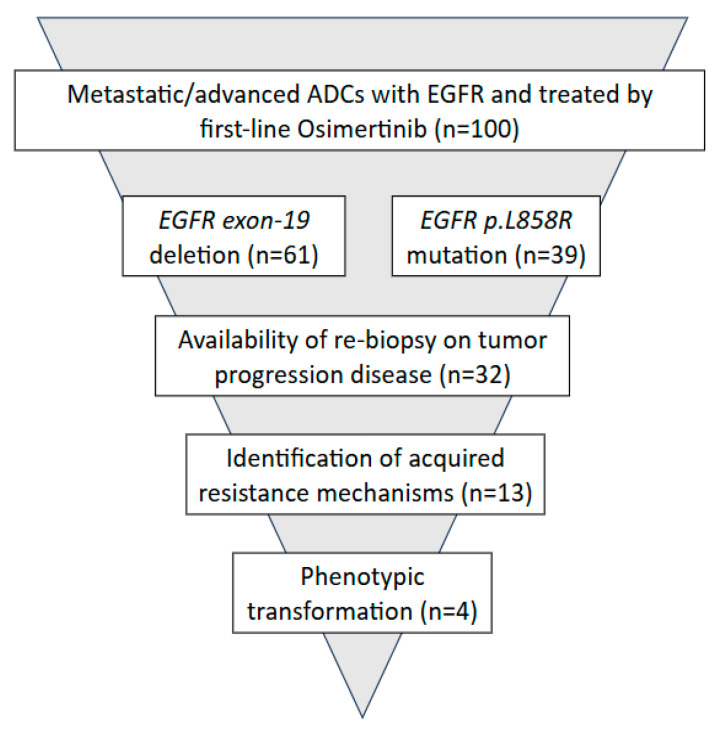
Study design.

As shown in Figure 1, 32 out of 100 cases of advanced and/or metastatic lung ADC underwent rebiopsy at the time of disease progression to first-line Osimertinib. Resistance to Osimertinib occurred after a median of 17 months and 12 months for patients with exon 19 deletions and p.(L858R) mutation, respectively. Among rebiopsied mADCs, in 41% of cases, known acquired resistance mechanisms were identified; out of these, 13% of cases underwent phenotypic transformation.

Out of the 22 rebiopsied cases for which *TP53* mutational status was available, nine patients (40.9%) harbored a pathogenic *TP53* variant in the diagnostic tumor specimens. Notably, *TP53* mutations were more frequently detected in association with *EGFR* exon-19 deletions. Further details regarding the rebiopsied cases are presented in Table 3.

### 2.4. Transformed Cases

**Figure 2:**
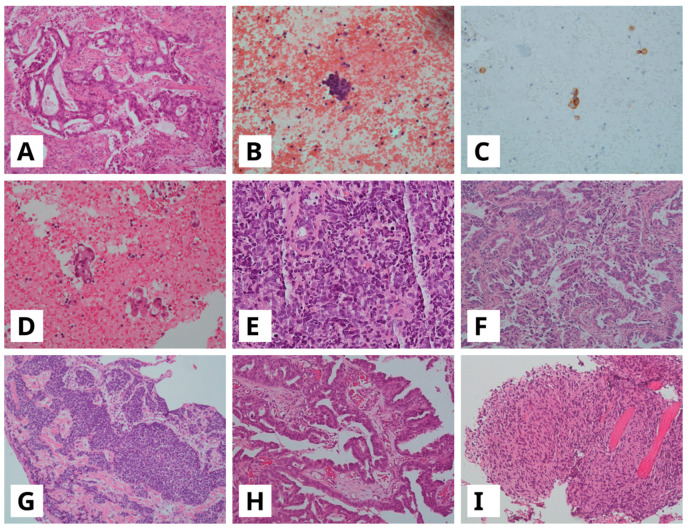
(**A**–**C**) Lung adenocarcinoma (ADC) in bronchial biopsy ((**A**), hematoxylin and eosin, H&E) showing transformation to small cell lung cancer (SCLC) on cell block from pleural effusion ((**B**), H&E) with strong cytoplasmatic immunoreactivity for synaptophysin (**C**); (**D**,**E**) ADC in CT-guided biopsy ((**D**), H&E) with following SCLC transformation on bronchial biopsy ((**E**), H&E); (**F**,**G**), ADC in pleural biopsy ((**F**), H&E), with SCLC transformation on bronchial biopsy ((**G**), H&E); (**H**,**I**), Lateral cervical lymph node with metastasis of lung ADC ((**H**), H&E) with following sarcomatoid carcinoma transformation on pleural biopsy ((**I**), H&E). All images are at a 10× magnification.

Below, we describe the four *EGFR* mutated cases showing histological transformation, hereafter named T1, T2, T3 and T4, at first-line progression to Osimertinib:

- T1: 58-year-old-woman, received Osimertinib for approximately 25 months. At progression, a histological transformation to SCLC was observed (Figure 2A–C). This case preserved *EGFR* exon-19 deletion on the tumor progression specimen. Furthermore, it harbored also a *TP53*-activating alteration c.776A>T p.(D259V) on exon 7, which was already detected at diagnosis. Our liquid biopsy, performed on the plasma sample at progression time, was negative. The patient received second-line treatment with carboplatin and etoposide chemotherapy combined with radiotherapy targeting a mediastinal metastatic lesion. After 14 months, the patient showed progression disease and was switched to topotecan chemotherapy for an additional 13 months. The patient died two months later.
- T2: A 61-year-old-woman developed histological transformation to SCLC after 14 months of Osimertinib therapy (Figure 2D,E). The tumor retained the EGFR exon 19 deletion and showed an acquired *MET* amplification at progression. Through examination of the diagnostic specimen, it was found that this case harbored a *TP53* pathogenic alteration c.329G>C p.(R110P) on exon 4. Liquid biopsy performed on the plasma sample at progression time confirmed *MET* amplification as an acquired resistance mechanism. According to the presence of *MET* amplification, the patient received an MET inhibitor, Tepotinib, in combination with Osimertinib as second-line treatment. After disease progression, five months later, she underwent carboplatin and etoposide chemotherapy combined with the PD-L1 inhibitor Atezolizumab. She continued treatment for 7 months before her death.
- T3: A 78-year-old-man with a histological transformation to SCLC after 14 months of Osimertinib therapy (Figure 2F,G). Through examination of the diagnostic specimen, it was found that this case harbored a *TP53*-activating alteration c.659A>G p.(Y220C) on exon 6. Liquid biopsy was not performed. Following disease progression, the patient received palliative care and died one month later.
- T4: A 72-year-old-woman developed transformation to spindle cell carcinoma, a variant of sarcomatoid carcinoma, after approximately 11 months of treatment (Figure 2H,I). The tumor harbored a *TP53*-activating alteration c.602del p. (L201fs), within exon 6, on the diagnostic specimen. Liquid biopsy at progression detected only the *EGFR* exon 19 deletion with no additional resistance mechanisms. The patient was treated with carboplatin combined with pemetrexed as second-line treatment chemotherapy but died three months later.

Further details about these cases are reported in Table 4.

## 3. Materials and Methods

### 3.1. Study Cohort and Design

The study included all consecutive cases of advanced and/or metastatic lung ADC harboring *EGFR* mutations diagnosed at the University Hospital of Pisa from January 2020 to December 2022. Histological and cytological diagnoses were performed by expert pathologists according to the WHO 2021 histological and immunohistochemical criteria [1]. Tumors were classified as advanced stages based on the latest American Joint Commission on Cancer—Tumor Nodes Metastasis (TNM) classification [42]. This study was conducted in accordance with the principles of the 1975 Helsinki Declaration. Written informed consent was obtained from all patients prior to tumor biopsy or surgical resection. All cases were anonymized for this study, and no sensitive data were used. This study did not interfere with routine clinical management.

### 3.2. Molecular Analysis

Molecular analyses were performed on formalin-fixed paraffin-embedded (FFPE) tissue samples from surgical resections, biopsies, and cell-blocks or on Papanicolaou-stained smears. For each case, the specimen with the highest tumor cell content was selected to determine the status of predictive biomarkers routinely tested for advanced NSCLC. DNA extraction from FFPE samples involved three 10 μm thick unstained sections that were processed by standard deparaffinization in xylene and graded ethanol rehydration. Cytologic smears underwent coverslip removal by incubation in xylene for 48 hours, which was followed by graded ethanol rehydration (99%, 95%, 70%, and 50%, 10 min each). All samples were enriched for cancer cells by manual macrodissection. DNA purification was carried out using the QIAamp DNA Mini Kit (Qiagen, Hilden, Germany) according to the manufacturer’s protocol. DNA concentration and integrity were assessed via spectrophotometry and a real-time PCR kit (Diatech Pharmacogenetics, Jesi, Italy). Molecular testing methodologies were performed according to changing practice patterns over time. Samples collected in 2020 were analyzed by MALDI-TOF technology on the Sequenom platform (Agena Bioscience, San Diego, CA, USA) using the kit Myriapod Lung Status (Diatech Pharmacogenetics, Jesi, Italy) according to the manufacturer’s instructions [43]. For samples collected from 2021 to 2022, next-generation sequencing (NGS) was performed with the amplicon-based panel Myriapod-NGS Cancer panel DNA (Diatech Pharmacogenetics, Jesi, Italy), according to the manufacturer’s protocol. Clinically relevant gene mutations within *EGFR*, *BRAF*, *KRAS* and *MET* genes were evaluated. *TP53* mutations in diagnostic tumor tissue were assessed by direct Sanger sequencing (primers listed in Supplementary Table S1).

Circulating cell-free DNA (cfDNA) was analyzed externally by digital real-time PCR from 4 mL of plasma.

Histopathological and clinical data were retrospectively reviewed and collected for each patient.

### 3.3. PD-L1 Evaluation

PDL-1 expression in tumor tissue was assessed by immunohistochemistry (IHC). A representative tissue block or cell block from each lesion was selected. Tissue sections of 4 μm thickness were deparaffinized in xylene, rehydrated using a graded series of ethanol solutions, and then subjected to immunohistochemical staining. IHC straining was performed using the rabbit monoclonal anti-PD-L1 antibody (clone SP263, Roche-Ventana, Oro Valley, AZ, USA) with the OptiView DAB IHC Detection Kit and OptiView Amplification Kit (Roche-Ventana, Oro Valley, AZ, USA). Immunostaining was performed as a fully automated assay using BenchMark XT automated slide stainers (Roche-Ventana, Oro Valley, AZ, USA). Negative controls were carried out by omitting the primary antibodies. Immunohistochemical evaluation was performed independently by two pathologists (G.A. and A.C. (Alessandra Celi)), blinded to clinicopathological characteristics and molecular data, as previously described. PD-L1 expression was quantified using the tumor proportional score (TPS), which was defined as the percentage of viable tumor cells exhibiting partial or complete membrane staining of any intensity among all of the viable tumor cells in the examined section [44].

### 3.4. Data Analysis

Within the study cohort, the prevalence and patterns of histological transformation were evaluated in *EGFR*-mutated patients undergoing rebiopsy at progression during first-line Osimertinib treatment. The persistence of *EGFR*-activating alterations and the coexistence of multiple resistance mechanisms on rebiopsy were evaluated.

## 4. Discussion

NSCLC is one of the most frequent and aggressive human cancers. Lung ADC is the most common histotype, and the majority of cases are diagnosed in advanced stages. In 15% of cases, lung ADCs have driver mutations in the *EGFR* gene, which are mainly in-frame deletions in exon 19 and the p.(L858R) point mutation in exon 21 [7]. These mutations are predictive of response to EGFR-TKIs. The third-generation EGFR-inhibitor Osimertinib, initially developed for patients with acquired resistance to 1st and 2nd-generation EGFR-TKI, is the election method for the first-line treatment [7]. However, acquired resistance inevitably occurs [8]. Mechanisms of resistance can consist of an on- target EGFR altered signaling pathway, off-target alterations and phenotypic transformation. The most common on-target resistance mechanism to first-line Osimertinib is the mutation of p.(C797S) within *EGFR* exon 20, which is observed in 22–44% of cases [8]. The *EGFR* p.(C797S) leads to drug resistance by breaking the covalent bond between the inhibitor and the mutant EGFR site [25]. On the other hand, the most frequently acquired off-target resistance mechanism is *MET* amplification (15% of cases). Alterations in *RET*, *ALK*, *BRAF*, *KRAS*, *PIK3CA* and *FGFR* genes have also been reported [8,45]. Another important mechanism of acquired resistance to first-line Osimertinib consists of histological transformation occurring in about 15% of cases [8], whose detection necessarily requires a tumor tissue rebiopsy. The most common type of histological transformation is represented by an ADC switch to SCLC with a prevalence of 3–14% [16]. Less common histological changes occur in 1.1–10% of cases and include transformation to SCC, sarcomatoid carcinoma and LCNEC [26,34,39].

In our study, from a consecutive series of 100 *EGFR* mutated ADCs, only 32 underwent tumor rebiopsy at progression time and 4 (12%) showed histological transformation. Although in agreement with the literature [8], the prevalence of histological transformation here reported could be underestimated. In fact, tumor rebiopsy at Osimertinib progression was performed in a minority of cases. In the clinical practice, tissue rebiopsy is not always feasible for several reasons, such as the accessibility of the metastatic site and the patients’ clinical conditions. Moreover, repeated rebiopsies can be associated with increased risks and costs [46].

Regarding histological transformations detected in our series, three cases switched to SCLC, and one switched to sarcomatoid carcinoma. A proper and correct histological diagnosis greatly influences each patient’s clinical outcome. For example, the transformed SCLCs are associated with a poor survival rate, and up to 60% of patients achieve consistent response rates with concurrent chemo-radiotherapy [1,47]. Several studies are evaluating the genetic background of transformed ADCs to understand whether there are molecular alterations acting as predisposing factors [10] potentially detectable also on liquid biopsy. Among the genetic alterations usually associated with phenotypic transformation, there are *TP53* pathogenic mutations [18,48]. Marcoux et al. found that approximately 3% to 10% of *EGFR*-mutant NSCLCs undergoing SCLC transformation often present concurrent *TP53* pathogenic mutations [11]. In *EGFR*-mutated ADCs, the co-occurrence of *TP53* pathogenic alterations has been reported to increase the risk of histological changes not only in SCLC transformation but also in SCC, sarcomatoid carcinoma and LCNET [30,37,40]. TP53 plays an important role in phenotypic plasticity [24] and interestingly, in our cohort, all transformed cases harbored *EGFR* exon-19 deletions and *TP53* co-mutations on tumor diagnosis. To deepen its role in histological transformation, ADCs characterized by this co-mutation should be further investigated both at molecular and clinical levels. In the absence of tumor tissue availability, liquid biopsy can be used to detect gene alterations in plasma; however, no molecular biomarkers suggestive of histological transformation have yet been identified [10]. Moreover, according to tumor volume, type and site, shedding levels can be different, and some molecular alterations can be missed by liquid biopsy analysis. In our study, liquid biopsies at progression time were available for three out of four transformed cases: one was negative, one presented only *EGFR* exon-19 deletion, and one presented only *MET* amplification. Probably negative cases can be due to the low amount of released cfDNA, and also intra-tumor heterogeneity can strongly impact this type of test [49].

In addition, resistance to Osimertinib can be extremely heterogeneous [9], and more molecular mechanisms can coexist. For instance, one of our reported cases harbored both *MET* amplification and histological transformation.

Although obtained on a limited series number, our data, in agreement with the available literature, suggest that at progression time, tumor rebiopsy should be considered whenever possible to better evaluate resistance mechanisms and to properly define therapeutic strategies.

## 5. Conclusions

Osimertinib is currently the preferred first-line treatment for *EGFR*-mutated NSCLCs; acquired resistance inevitably occurs. Histological transformation has been widely reported as an acquired, off-target resistance mechanism. While liquid biopsy represents a valuable tool for detecting *EGFR* resistance mechanisms, only a pathological examination of tumor rebiopsy can reliably identify phenotypic transformation, which strongly influences therapy assessment and prognosis.

## Figures and Tables

**Table 1:** Characteristics of described *EGFR*-mutated lung ADCs transformed to SCLC under Osimertinib treatment.

Authors	Year	Median Age	Median Transformation Time (Months)	Co-Occurred Gene Alterations
Hao et al. [17]	2023	65	17.8	*TP53*, *RBP1*, *SOX2*
Lai et al. [20]	2021	48	12	-
Po-Hsin Lee et al. [21]	2022	55	16	-
Bingnan Zhang et al. [22]	2022	58	24	*TP53*, *RBP1*, *PIK3CA*, *MYC*, *MET*, *BRAF*
Isa Mambetsariev et al. [23]	2022	60	16	*TP53*, *RBP1*
Cai Bao Jin et al. [24]	2021	60	15	-
Chen et al. [16]	2023	58	33	*TP53*, *RB1*
Ferrer et al. [25]	2019	61	16	*PI3K*, *c-MET*, *ALK*

**Table 2:** EGFR mutations in the study cohort.

EGFR Alterations	Number of Cases
EGFR Exon-19 deletions	61
p.(E746_A750del)	35
p.(E746_T751del)	2
p.(L747_E749del)	1
p.(L747_P753delinsS)	1
p.(E746_S753delinsV)	1
p.(E746_T751delinsL)	1
p.(L747_P753>S/p.(L747_T751del)	5
p.(E746_A750>QP)/p.(E746_S752del)	4
p.(L747_A750>P/L747_S752del)	2
p.(E746_A750>QP)/p.(E746_S752del)	1
Del19 *	8
EGFR p.(L858R) mutation	39
p.(L858R)	36
p.(L858R) + p.(S768I)	1
p.(L858R) + p.(A871G)	1
p.(L858R) + p.(V834L)	1

* The presence of *EGFR* exon-19 deletion was identified by using a real-time PCR assay not able to characterize the specific alteration type.

**Table 3:** Acquired resistance mechanisms identified in this study cohort.

INFO	DIAGNOSIS				PROGRESSION		
Case ID	Sample Type, Site	EGFR	PD-L1	TP53	Sample Type, Site	Time (Months)	Identified Resistance
1	Citology, lung	p.L747_p753>S/p.(L747_T751delREAT) *	NA	NA	Citology, pleural liquid	6	MET amplification (ratio 3.7; CNV 7.6)
2	Histology, lung	p.(E746_A750del)	2%	p.(D259V)	Histology, lung	36	Histological transformation
3	Histology, lung	p.(E746_A750del)	NEG	p.(Y220C)	Citology, liquid pleuritic	15	Histological transformation
4	Histology, lung	p.(E746_A750delELREA)	1%	wt	Histology, lung	13	NO
5	Histology, lung	Exon 19 deletion **	NEG	p.(Q165Ter)	Citology, lung	38	NO
6	Histology, lung	p.(E746_A750del)	45%	wt	Citology, lung	13	NO
7	Cytology, lung	p.(L858R)	10%	wt	Citology, liquid pleuritic	23	
8	Histology, lung	p.(L858R)	20%	NA	Histology, lung	6	KRAS p.(G12D)
9	Citology, lung	p.(L858R)	20%	wt	Histology, lung	12	NO
10	Histology, lung	p.(E746_A750>QP)/p.(E746_S752delELREATS) *	95%	NA	Histology, lung	18	NO
11	Citology, liquid pleuritic	p.(E746_A750del)	70%	p.(Leu201fs)	Histology, lung	11	Histological transformation
12	Histology, lung	p.(E746_T751delinsL)	NEG	NA	Citology, liquid pleuritic	15	NO
13	Histology, lung	p.(L858R)	10%	NA	Citology, liquid pleuritic	5	NO
14	Histology, lung	p.(L858R)	NEG	wt	Histology, lung	12	NO
15	Histology, lung	p.(L858R) + p.(V834L)	30%	p.(I232S)	Histology, lung	24	NO
16	NA	p.(L747_S752delinsP)	NA	NA	Histology, lung	20	NO
17	Histology, lung	p.(E746_A750del)	3%	NA	Citology, liquid pleuritic	10	NO
18	Histology, lung	p.(E746_A750del)	5%	wt	Citology, liquid pleuritic	11	KRAS p.(G13D)
19	Citology, lung	p.(E746_A750del)	1%	wt	Citology, lung	2	NO
20	Histology, lung	p.(E746_A750del)	NEG	p.(R273C)	Histology, lung	12	NO
21	Histology, lung	p.(E746_A750del)	10%	NA	Histology, lung	15	EGFR EX20 p.(C797S)
22	Histology, lung	p.(E746_A750del)	15%	wt	Histology, lung	12	MET AMPL POS (ratio 6.2; CNV 12.1)
23	Citology, lung	p.(L858R)	1%	wt	Citology, lung	5	NO
24	Histology, lung	p.(E746_A750del)	NA	p.(R110P)	Histology, lung	14	Histological transformation, MET amplification (liquid biopsy)
25	Biopsy, lung	p.(E746_A750del)	NA	NA	Histology, lung	18	NO
26	Biopsy, lung	p.(E746_A750del)	0.7	p.(V274D)	Histology, lung	10	MET amplification
27	Biopsy, sacral lesion	p.(L747_P7563>S/p.(L747_t751delLREAT) *	Neg	wt	Histology, lung	9	NO
28	Biopsy,	p.(L858R)	Neg	wt	Citology, liquid pleuritic	11	NO
29	Citology, lung	p.(E746_A750del)	0.01	NA	Histology, lung	36	EGFR EX20 c.2389T>A p.(C797S)
30	Biopsy, lung	p.(L858R) + p.(A871G)		NA	Histology, lung	NA ^&^	NO
31	Biopsy, lung	p.(E746_A750del)	Neg	wt	Histology, lung	48	NO
32	Citology, lung	p.(E746_A750del)	0.7	p.(C135Y)	Histology, lung	7	NO

* The employed diagnostic test was not able to discriminate between these types of *EGFR* exon-19 deletions. ** The presence of an *EGFR* exon-19 deletion was identified by using a real-time PCR assay not able to characterize the specific alteration type. ^&^ NA: not available data.

**Table 4:** Details of histological transformed cases.

Transformed Cases	T1	T2	T3	T4
**Diagnosis**				
Tumor histology	ADC	ADC	ADC	ADC
Sample type, site	Biopsy, lung	Biopsy, pleura	Biopsy, lymph node	Citology, pleural effusion
EGFR del19	p.(E746_A750del)	p.(E746_A750del)	p.(E746_A750del)	p.(E746_A750del)
PD-L1	2%	Not available	0%	70%
1-line TKI time	25 months	14 months	14 months	11 months
**Progression**				
Tumor histology	SCLC	SCLC	SCLC	Spindle cell
Sample type, site	Histology, lymph node	Biopsy, lung	Citology, pleural effusion	Biopsy, lung
EGFR del19	p.(E746_A750del)	p.(E746_A750del)	p.(E746_A750del)	p.(E746_A750del)
PD-L1	5%	Not available	Neg	25%
Concurrent resistance mechanisms	Not identified	MET amplification	Not identified	Not identified
Liquid biopsy	Neg	Not executed	MET amplification	Exon 19 deletion

## Data Availability

The original contributions presented in this study are included in the article/Supplementary Materials. Further inquiries can be directed to the corresponding authors.

## Supplementary Materials

The following supporting information can be downloaded at https://www.mdpi.com/article/10.3390/ijms262110462/s1.

## Abbreviations

The following abbreviations are used in this manuscript:

mADC	metastatic lung adenocarcinoma
NSCLC	non-small cell lung cancer
SCC	squamous cell carcinoma
LCNEC	large cell neuroendocrine carcinoma
TKI	tyrosine kinase inhibitors
SCLC	small cell lung cancer
tSCLC	transformed SCLC
tLCNET	transformed LCNEC
EMT	epithelial–mesenchymal transition
H&E	hematoxylin and eosin
FFPE	formalin-fixed paraffin-embedded
IHC	immunohistochemistry
TPS	tumor proportional score
